# Development of Flow State Self-Regulation Skills and Coping With Musical Performance Anxiety: Design and Evaluation of an Electronically Implemented Psychological Program

**DOI:** 10.3389/fpsyg.2022.899621

**Published:** 2022-06-17

**Authors:** Laura Moral-Bofill, Andrés López de la Llave, Mᵃ Carmen Pérez-Llantada, Francisco Pablo Holgado-Tello

**Affiliations:** Department of Methodology of the Behavioral Sciences, Universidad Nacional de Educación a Distancia, Madrid, Spain

**Keywords:** Flow state, performing musicians, Musical Performance Anxiety, social skills, electronic intervention program, Flow experience

## Abstract

Positive Psychology has turned its attention to the study of emotions in a scientific and rigorous way. Particularly, to how emotions influence people’s health, performance, or their overall life satisfaction. Within this trend, Flow theory has established a theoretical framework that helps to promote the Flow experience. Flow state, or optimal experience, is a mental state of high concentration and enjoyment that, due to its characteristics, has been considered desirable for the development of the performing activity of performing musicians. Musicians are a population prone to health problems, both psychological and physical, owing to different stressors of their training and professional activity. One of the most common problems is Musical Performance Anxiety. In this investigation, an electronic intervention program was carried out for the development of psychological self-regulation skills whose main objective was to trigger the Flow response in performing musicians and the coping mechanism for Musical Performance Anxiety. A quasi-experimental design was used with a control group in which pre- and post-measures of Flow State, Musical Performance Anxiety and, also, Social Skills were taken. Sixty-two performing musicians from different music colleges in Spain participated in the program. Results indicated that the intervention significantly improved Flow State (*t* = –2.41, *p* = 0.02, *d* = 0.36), and Sense of Control (*t* = –2.48, *p* = 0.02, *d* = 0.47), and decreased Music Performance Anxiety (*t* = 2.64, *p* = 0.01, *d* = 0.24), and self-consciousness (*t* = –3.66, *p* = 0.00, *d* = 0.70) of the participants in the EG but not CG. The changes in the EG after the program showed the inverse relationship between Flow and Anxiety. Two important theoretical factors of both variables (especially in situations of performance and public exposure), such as worry and the feeling of lack of control, could be involved. The results are under discussion and future lines of research are proposed.

## Introduction

From the Positive Psychology approach, the study of emotions has been addressed to try to understand how they influence people’s health, performance, or their overall life satisfaction (Seligman, 2008).

In a review of 12 school-based interventions to foster student well-being and academic performance, following a Positive Psychology approach, it was found that implemented programs were consistently related to student well-being, social relationships, and academic performance (Waters, 2011). In the specific field of music, an attempt has been made to understand how professional musicians experience well-being in the light of Positive Psychology (Ascenso et al., 2017).

Framed in Positive Psychology, Flow theory (Csikszentmihalyi, 1975, 1990; Csikszentmihalyi and Csikszentmihalyi, 1988; Csíkszentmihályi, 1997) has established a framework that, specifically in the field of sport, has contributed to developing the psychological skills of athletes to optimize their enjoyment and performance (Jackson and Csikszentmihalyi, 1999; Norsworthy et al., 2017; Jackman et al., 2019).

In the same line of thought, for a few years now, the need for musicians to develop self-regulation skills has been considered, which would complete an education focused on eminently technical-performance aspects (Brodsky, 1996; Williamon, 2004; Clark and Williamon, 2011; Wrigley and Emmerson, 2013; Cohen and Bodner, 2019a; Moral-Bofill et al., 2022).

It has been noted how musical performance poses multiple simultaneous demands at the cognitive (Kenny and Osborne, 2006), affective (Kenny, 2005), conative, and kinesthetic (Altenmüller et al., 2000), and motor system (Kenny and Ackermann, 2015) levels. In fact, performing musicians are exposed to a relatively high risk of physical and psychological stress that can lead to disorders and health problems. In order to cope better with all these demands (physical, psychological, and social), the need to implement appropriate interventions has been suggested to help make their musical career rewarding and steady over time (Kenny and Ackermann, 2016).

Recent studies have suggested that the psychosocial work environment of musicians may be considered more demanding than that of other occupations (Holst et al., 2012; Burak and Atabek, 2019; Détári et al., 2020; Musgrave and Gross, 2020). For example, Vaag et al. (2016) reported that, compared to the general population, professional musicians had more symptoms of anxiety and depression. It also appears that music students show a greater number of these symptoms compared to the general student population (Spahn et al., 2004; Vaag et al., 2021), in addition to lower levels of self-efficacy and self-regulation (Ginsborg et al., 2009), and psychosocial well-being (Panebianco-Warrens et al., 2015). Anxiety and depression are not only highly prevalent among music students, but their symptom burden is even higher than that seen among professional musicians (Kegelaers et al., 2021).

One of the most common and specific problems of performing musicians is Musical Performance Anxiety (MPA). MPA is the experience of strong and persistent anxious apprehension related to a musical performance, which manifests as a combination of affective, cognitive, somatic, and behavioral symptoms. It is triggered in different performance contexts, but it is more intense when (a) the performer is overly concerned about their image, (b) there is fear of evaluation and judgment from others, and (c) there is a fear of making mistakes. Although it can be specific, focused on musical performance, it can also occur alongside other anxiety disorders, for example, social anxiety disorder (Kenny, 2011). Numerous studies show how MPA can affect musicians of any age and at any stage of their education or career (Fishbein et al., 1988; Ryan, 2005; Kenny D. et al., 2014). In fact, musicians under 30 have a higher risk of experiencing it (Kenny D. et al., 2014). This could be related to the lower level of performing experience of performing musicians in college (Biasutti and Concina, 2014). Students, therefore, may suffer from MPA in addition to experiencing periods of burnout (Bernhard, 2010) and considering continuing as performing musicians (Fehm and Schmidt, 2006; Osborne, 2016). It is also necessary to mention that gender is an important factor related to MPA. Numerous studies have found that there is a higher prevalence and intensity of MPA in women than in men (cf. Burin and Osorio, 2017).

On the other hand, it has been suggested that anxiety has a negative relationship with a Flow state (Csikszentmihalyi, 1975). This has led to the suggestion that interventions to promote a Flow state could contribute to the reduction of MPA and facilitate musical performance (Lamont, 2012; Wrigley and Emmerson, 2013; Iusca, 2015; Cohen and Bodner, 2019a). In fact, studies have been published that have found negative correlations between MPA and Flow in musicians (Kirchner et al., 2008; Fullagar et al., 2013; Stocking, 2013; Cohen and Bodner, 2019a; Moral-Bofill et al., 2022).

Flow State (FS) is a subjective state of mind in which a person is involved in what they are doing, highly focused, carefree, and with a positive emotion of gratification (Csikszentmihalyi, 1990). People describe FS in the same way, regardless of culture, social class, gender, or different fields of activity, such as work and leisure. Research has identified the different components involved in Flow. These components are usually conceptualized as those elements that phenomenologically configure FS and the factors that are considered to be the conditions for that FS to occur (Nakamura and Csikszentmihalyi, 2009). With regard to these previous conditions, the following have been noted: (1) There is a balance between the skills and the challenge to be faced (balance). (2) Having clear goals (goals). (3) Receiving clear feedback on how the activity is progressing (feedback). Whereas, the six components that characterize FS would be: (1) Concentration on the task (concentration): describes the intense and focused concentration on the present moment. (2) Merging of awareness and action (merging): reflects a feeling of acting effortlessly, with a deep involvement that removes awareness of the concerns and pressures of daily life. (3) Loss of self-consciousness (consciousness): manifests the decrease and/or disappearance of self-consciousness as a social actor. (4) Sense of control (control): feeling like oneself can control one’s actions and can cope with the situation. (5) Transformation of time (time): having the feeling that time has passed in a different way (e.g., faster, or slower than normal). (6) Autotelic experience (autotelic): the activity is experienced as intrinsically rewarding, a matter that establishes the highly positive emotional value of this experience.

It has been noted that it is important to properly operationalize FS as an optimal state of consciousness that is relatively rare in daily life, intrinsically rewarding and differentiated from the conditions that cause it (balance, goals, and feedback) (Abuhamdeh, 2020).

Flow state has been investigated in different fields. In the field of Sports Psychology, it is a widely researched construct and the works of Susan Jackson have been considered as a reference (Jackson and Eklund, 2002, 2004). But it has also been investigated in other areas, such as work (Csikszentmihalyi and Lefevre, 1989; Bryce and Haworth, 2002; Eisenberger et al., 2005; Peifer and Wolters, 2021), education (Carli et al., 1988; Bakker, 2005; Rathunde and Csikszentmihalyi, 2005), creativity (Csikszentmihalyi, 1996, 2006; Csikszentmihalyi and Rich, 1998), leisure (Lefevre, 1988; Schallberger and Pfister, 2001), the arts (cf. Harmat et al., 2021), human–computer interaction (Triberti et al., 2021), or recently in high capacities (Moral-Bofill et al., 2020a). The field of music psychology has not been a stranger to this interest (cf. Chirico et al., 2015; Tan and Sin, 2021), more and more studies are being found in the field of music education and/or related to musical performance (Custodero, 2002, 2005; Fritz and Avsec, 2007; Sinnamon et al., 2012; Fullagar et al., 2013; Marin and Bhattacharya, 2013; Wrigley and Emmerson, 2013; Iusca, 2015; Cohen and Bodner, 2019a,b; Moral-Bofill et al., 2020b). In educational and training contexts, it is considered a source of motivation that can promote learning and the development of skills over time (Nakamura and Csikszentmihalyi, 2009). In the field of music, it has been considered a rewarding and motivating experience that promotes the desire to continue doing the activity that is being carried out (Custodero, 2002, 2005). It has been linked to creativity, enhancing creative composition activities (Byrne et al., 2003; MacDonald et al., 2006). In the case of performing musicians, it has been suggested that it may contribute to greater enjoyment of the performing activity and a reduction in MPA (Kirchner et al., 2008; Sinnamon et al., 2012; Fullagar et al., 2013; Wrigley and Emmerson, 2013; Iusca, 2015; Cohen and Bodner, 2019a,b; Moral-Bofill et al., 2020b); as well as the musicians continuing to be involved in music (Woody and McPherson, 2010).

Finding FS relationships with other variables appears as a research area that can help to understand ways to promote FS. According to Flow theory, there is the concept of “autotelic” personality which suggests that certain personal characteristics may represent a greater disposition to undergo the Flow experience (Csikszentmihalyi, 1990). There are studies that have shown that Flow has a negative relationship with neuroticism and a positive relationship with responsibility, but not with intelligence (Ullén et al., 2012). It has also been suggested that people with a high internal locus of control scores may enjoy the activity more when faced with challenges and reach FSs more easily (Keller and Blomann, 2008; Mosing et al., 2012). But also, the need for achievement (Eisenberger et al., 2005), mental tenacity (Crust and Swann, 2013), self-control (Kuhnle et al., 2012), the quest for novelty, and persistence (Teng, 2011), have shown positive relationships with Flow.

A systematic review has also shown that there is a small to moderate relationship between FS and performance. The results show that such a relationship is consistent in both games and sports activities (Harris et al., 2021).

Other factors related to FS are emotions. FS is related to a positive emotional state and is one of the factors that has been identified as a determinant of subjective well-being (Csikszentmihalyi, 1990). In music students, positive relationships have been found between the positive emotional aspects of subjective well-being and the predisposition for Flow (Fritz and Avsec, 2007). Both the hours dedicated to practice and emotional intelligence, evaluated with self-report measures, have been shown to be predictors of Flow in pianists (Marin and Bhattacharya, 2013).

In fact, Flow theory provides a framework to promote a more positive and satisfying relationship with performing an activity and preventing MPA. Most forms of performance anxiety are difficult to treat, and a person’s anxiety level after undergoing treatment is rarely reduced to the anxiety levels experienced by non-anxious people (Kenny, 2005). For this reason, the best ways to address the problem of MPA would be, on the one hand, to prevent its occurrence (Spahn, 2015; Kenny and Ackermann, 2016) and, on the other, implementing positive coping strategies in an educational context, from very early on (Spahn, 2015).

Alongside the research that has focused on identifying the problems that occur in the field of musicians’ health, there has been an increased focus on the prevention of these problems and the promotion of artists’ health during their musical and artistic education (Araújo et al., 2017; Perkins et al., 2017; Matei et al., 2018; Aalberg et al., 2019). Prior to this focus on health promotion and prevention of disorders or health problems, there is a significant volume of research that has focused on trying to find solutions for MPA. Cognitive behavioral therapy (CBT) is the one with the greatest scientific support in terms of its efficacy in treating it (Kenny, 2011). In the case of severe MPA (with panic and depression), short-term intensive dynamic psychotherapy has shown more promising results (Kenny D. T. et al., 2014; Kenny, 2016; Kenny et al., 2016). Mention should also be made of treatment with beta-blockers, which are used, according to studies, by up to 31% of professional orchestral musicians to reduce symptoms of physiological arousal associated with MPA (Kenny D. et al., 2014).

Other interventions for the treatment of MPA have also been studied, such as biofeedback (Egner and Gruzelier, 2003; Thurber et al., 2010), yoga (Khalsa et al., 2013), the Alexander technique (cf. Klein et al., 2014), or guided imagery with progressive muscle relaxation (Kim, 2008); however, the evidence for this type of intervention is lower and there are significant methodological limitations (Burin and Osorio, 2017; Juncos and de Paiva e Pona, 2018).

Recently, research has been presented that has studied the effects of psychological interventions for the treatment of MPA such as acceptance and commitment therapy (Juncos and de Paiva e Pona, 2018; Clarke et al., 2020; Shaw et al., 2020) or expressive writing (Tang and Ryan, 2020). These studies show satisfactory results and may be the beginning of a new approach to dealing with MPA, but, for now, they are also case studies or have very small samples, so further research is necessary.

Within an approach that is more focused on the prevention of MPA problems and the promotion of adaptive coping habits for this problem, and due to the many common characteristics of sports and musical performances, research has been presented that studies the effects of specific intervention programs. These interventions place an emphasis on psychological techniques such as mental rehearsal, goal setting, focusing on strengths, with the ultimate goal of reaching FS or a full immersion experience in a performance (Williams, 2010). Some intervention studies that integrate sport and performance psychology methods have shown improvements in reducing MPA (Clark and Williamon, 2011; Hoffman and Hanrahan, 2012; Osborne et al., 2014; Braden et al., 2015; Cohen and Bodner, 2019b) as well as in musical performance (Hoffman and Hanrahan, 2012; Cohen and Bodner, 2019b). However, these studies did not record Flow experience measures, with the exception of Cohen and Bodner (2019b) who took Flow measures with the “Dispositional Flow Scale-2” short scale (Martin and Jackson, 2008). The results of the intervention showed a statistically significant reduction in MPA. However, no differences were observed in the measures of Flow. However, in a recent study (Moral-Bofill et al., 2022) involving 139 performing musicians (college students and professionals) the results of the regression analysis showed that some of the same variables predicted both FS and MPA, while other variables predicted either FS or MPA (e.g., the variable Social Skills predicted both FS and MPA; Gender MPA; or Clear Study and Interpretive Objectives FS). In addition, they suggested that the musicians’ motivation and determination to develop their musical career could partially influence the FS experience, although it doesn’t necessarily affect the MPA levels (Moral-Bofill et al., 2022). Therefore, it is possible that interventions aimed at increasing FS may need to develop specific strategies aimed at that goal.

One challenge in evaluating the effects of intervention programs for the development of psychological skills for performance is the difficulty of implementing these programs over a long period of time and that, in addition, they are linked to the curricular activities of schools. The results of the analysis of a vast amount of research in the field of socio-emotional learning (SEL) in compulsory education, indicate that for effective learning of self-regulation skills, regular and consistent practice of these skills is necessary and, furthermore, that involvement of different educational strata is needed for optimal implementation (Durlak et al., 2011; Taylor et al., 2017). According to Bisquerra (2006), the importance of emotional education, in addition to the field of “formal education,” has spread to any life stage and also to different contexts. On the other hand, advances in neuroscience justify the need to consider emotions in education (Zull, 2006; Carew and Magsamen, 2010; Dolcos et al., 2011; Jung et al., 2014; Seli et al., 2016; Vogel and Schwabe, 2016; Tyng et al., 2017), not least because they are involved in self-regulation and attention control, and, in general, the development of cognitive skills. Also, from the multifaceted approach to intelligence, the importance of each and every intelligence needing adequate stimulation for their learning is noted. Personal intelligence (Gardner, 1983) too, since understanding and regulating emotional aspects, like other information processing capabilities, are learned. The implications of this argument are very relevant since it justifies the implementation of emotional education or SEL programs. But also, for these to be effective, they must be regular and consistent. This means that the curriculum has to consider dedicating regular hours to explicit learning of emotional skills development (Moral-Bofill et al., 2015). There are also results from mindfulness studies that show that regular practice is essential for promoting positive emotions, improving concentration and states of physiological relaxation (Davidson et al., 2003). Mindfulness develops and deepens over time, but requires a continuous commitment to its practice (Kabat-Zinn, 2003; Davidson, 2010).

A recent article has reviewed the benefits of integrating various approaches to MPA prevention or treatment and how it can contribute to the musician’s psychological training and performance preparation process. In this article, the contributions of emotion psychology and emotional regulation are shown as key elements for an appropriate approach to the MPA phenomenon, without forgetting the role of clinical psychology, performance psychology, and positive psychology (Kaleńska-Rodzaj, 2021).

Another recent study has found a strong relationship between MPA, social anxiety, and perfectionism, suggesting that some musicians with MPA also show symptoms of a co-morbid social phobia that is not specifically related to performance (Dobos et al., 2019). Evidence of this co-morbidity between social phobia (and other anxiety disorders) and MPA has also been shown in previous studies (Kenny, 2010).

Supporting this relationship with social anxiety, a recent study has found that social avoidance is among the set of MPA predictors (Lupiáñez et al., 2021). In fact, according to some studies, social skills are part of the necessary skill set for the performance of musicians (Kemp, 1996; Gaunt and Hallam, 2009). A recent study has found that both FS and MPA show statistically significant relationships (positive and negative, respectively) with social skills (Moral-Bofill et al., 2022). It has also been found that social relationships are a central aspect for musicians, and being successful in them presents a challenge both in the work and personal context, so it is suggested that social skills training is important in the context of professional music (Ascenso et al., 2017).

Previous studies show the importance of interpersonal relationships and social skills for performing musicians, as well as associations between MPA and social anxiety. However, interventions carried out to address the problem of MPA tend to focus on aspects of the individual (such as mental training, exposure, internal dialogue, activation control, etc.) and are generally focused on performance. A broader perspective would be for interventions to implement strategies for musicians to develop emotional and social awareness and regulation skills beyond specific performance preparation.

On another note, Internet use has expanded the way mental health interventions are implemented (Botella et al., 2009). Findings from different studies suggest an emerging evidence base supporting web-based mental health to support or treat a wide variety of mental disorders (Andersson and Titov, 2014; Lal and Adair, 2014); such as post-traumatic stress disorder (Kuester et al., 2016; Kuhn et al., 2017; Simblett et al., 2017), dissociative disorders (Brand et al., 2019; Fung et al., 2020), schizophrenia (Rotondi et al., 2010), anxiety (Reger and Gahm, 2009), or depressive symptoms (Karyotaki et al., 2021). Internet-based interventions have been found to be effective in reducing (mainly in adults) the symptoms of the most common mental disorders such as depression, anxiety, substance abuse, and eating disorders. However, more efforts are needed to implement and evaluate these types of programs in other contexts (Taylor et al., 2021). For example, evidence in the youth population is limited and further research and program development is needed (Reyes-Portillo et al., 2014). In any case, the results show that they are a promising resource for the psychological treatment of depression (Andersson and Cuijpers, 2009) and anxiety (Penate and Fumero, 2016). In addition, they improve even more when combined with some type of contact with the therapist. Notwithstanding, the disadvantage is that a higher dropout rate is noted (Penate and Fumero, 2016). Furthermore, it has been shown that they can be effective in managing stress in adults (Heber et al., 2017), in university students (Frazier et al., 2015) and in employees (Heber et al., 2016). In higher education students, a study differentiated between mental health prevention treatments aimed at students without a specific diagnosis compared to those aimed at students with a mild or moderate disorder. The results showed that the skills training interventions obtained medium and statistically significant effect sizes in both types of intervention. In addition, those interventions aimed at students with a specific diagnosis obtained better results when the participants had access to some type of support, face-to-face or online (Conley et al., 2016). Other results with university students showed that interventions designed from modules for the development of skills (promotion) can have a significant impact on the mental health of adolescents; however, more studies are needed to support this. On the other hand, the results of interventions aimed at prevention showed a statistically significant positive effect of CBT on symptoms of anxiety and depression in adolescents and young adults. In addition, the results suggested that face-to-face and/or online support for participants was an important feature for program completion and program outcomes (Clarke et al., 2015). Another systematic review showed that Internet interventions for mental health had small to moderate statistically significant effects on a range of conditions (depression, anxiety, stress, sleep problems, and eating disorders) but not on well-being. However, it is suggested that more research is needed to determine which interventions are most effective for different groups of students and to explore ways to increase treatment effectiveness (Harrer et al., 2019). Specifically, in the context of musicians, Ingle (2014) evaluated the effectiveness of an Internet-based health promotion program targeting Australian elite music students. A combined in-person and online learning program has also been carried out, with the aim of increasing self-efficacy levels in adolescent students through training in psychological skills for performance (Gill, 2019).

Furthermore, for the first time, the global impact of the COVID-19 pandemic has increased the demand for mental health services, and internet-based interventions may be particularly suitable for this purpose (Brog et al., 2022). For the same reason, as a consequence of COVID-19, schools have implemented many Internet-based educational platforms (Okmawati, 2020). Google Classroom is one of the technologies used (Sharda and Bajpai, 2021), also in university education (Gour, 2018). Probably due to its easy access and its free form, Google classroom is the most used worldwide (Ríos-Lozada et al., 2022). The results on its use suggest that it is an efficient and functional tool (Gour, 2018; Okmawati, 2020; Sharda and Bajpai, 2021), and it is perceived as a comfortable and easy-to-use technology (Santos, 2021).

The main objective of this research was to evaluate the effects of a program designed to promote FS and deal with MPA through the development of self-regulation skills. The program was aimed at a group of students and professors from a music conservatoire who are active performing musicians. Two dependent variables were considered: (a) FS and (b) MPA. As a secondary objective, the program’s effects on the variable Social Skills (SS) were studied.

The following hypotheses were made:

(1) The existence of significant differences in FS between the control group and the experimental group (EG) will be determined.
(2) The existence of significant differences in MPA between the control group and the EG will be determined.
(3) The existence of significant differences in SS between the control group and the EG will be determined.
(4) Significant differences in any of the variables considered in the control group will not be determined.

## Materials and Methods

### Research Design

This research was carried out using a quasi-experimental design that use both control groups and pretests. Specifically, we used the untreated control group design with dependents pretest and posttest sample. This design is frequently called the nonequivalent comparison group design, that is, may be, the most common of all quasi-experiments (Shadish et al., 2002).

### Participants

Senior music students or active performing teachers who had an internet-connected device and a Gmail email account were able to apply to participate in this program. They also had to consent to the research and agree to a declaration of commitment and sincerity to the program (see Supplementary Material 1). In addition to these requirements, the criteria for inclusion in the study were as follows: (a) being of legal age, (b) participation in at least 80% of the program’s activities, and (c) adequate completion of the measuring instruments applied over the duration of the program (pre-post). In the case of the control group, the last of the listed criteria and being of legal age were applied. Of the initial 142 participants who were enrolled, 80 did not meet the criteria for inclusion in the study, so ultimately 62 performing musicians participated in the research. Of these, 50 were students at the music conservatoire and 12 were vocational or higher education teachers. The age bracket ranged from 18 to 61 (*m* = 27.58 and *de* = 10.56). 32% (*n* = 20) were men (mean age = 30.25 and *DE* = 10.94) and 68% (*n* = 42) were women (mean age = 26.31 and *DE* = 10.26). The participants were divided into two groups based on the information collected in the form they completed and where the requirements for participation in the program had been explained. The participants who met all the requirements and agreed with the commitment of participation were part of the EG. Of the rest of the participants, those who met the basic requirements of being of legal age and performing musicians (senior students or active performing teachers), and agreed to the commitment to respond to the forms 3 months later, were part of the control group (CG). The rest of the participants were excluded from the research. EG consisted of *N* = 28 (9 men, mean age = 28.33 and *DE* = 11.57 and 19 women, mean age = 27.63 and *DE* = 12.57). CG was made up of *N* = 34 (11 men, mean age = 31.82 and SD = 10.69 and 23 women, mean age = 25.22 and *SD* = 8.01). Table 1 shows the percentages of participants based on the categorical variables collected for each group and for the total number of participants.

**TABLE 1:** Percentage of total participants and by groups according to the categorial variables considered.

Variable	Categories	% Total (*N* = 62)	% GE (*n* = 28)	% GC (*n* = 32)
Gender				
	Men (*n* = 20)	32.3%	32.1%	32.4%
	Women (*n* = 42)	67.7%	67.9%	67.6%
Work				
	Student (*n* = 50)	80.6%	67.9%	91.17%
	Professor (*n* = 12)	19.4%	32.1%	8.8%
Musical style				
	Classical (*n* = 57)	91.9%	96.4%	88.2%
	Other (*n* = 5)	8.1%	3.6%	11.76%
Musical instrument				
	Woodwind (*n* = 13)	21%	7.1%	32.4%
	Piano (*n* = 11)	17.7%	25%	11.8%
	Singer (*n* = 12)	19.4%	14.3%	23.5%
	Strings (*n* = 17)	27.4 %	35.7 %	20.6 %
	Other (*n* = 9)	14.4%	17.9%	11.8%

### Instruments

- Flow State Scale for Musical Performers (EFIM in its Spanish acronym) (Moral-Bofill et al., 2020b). This is a 24-item questionnaire that measures FS. It consists of six scales, each composed of conceptually different items. The scales covered by this instrument are as follows: action-awareness merging; concentration on the task; sense of control; loss of self-consciousness; transformation of time; and autotelic experience. To assess the degree of agreement with the formulation of each item, a Likert scale from 0 to 10 points is used, where 0 is strongly disagree and 10 is strongly agree. The scores for each of the six scales can be obtained separately, as well as overall FS. To respond to the EFIM scale, you must first specify the situation that is referenced to answer. The most appropriate time to answer the questions presented by this tool is at the end of the activity proposed as a criterion (Moral-Bofill et al., 2020b). The present research called for the situation to be a concert or public audition situation. Rates of reliability with Cronbach’s Alpha are greater than 0.80 on all scales and 0.92 for the FS global scale.
- KMPAI-E (Arnáiz, 2015), is the Spanish adaptation of the Kenny Music Performance Anxiety Inventory, K-MPAI (Kenny, 2009, 2011). The KMPAI-E is constructed of 40 items encompassing the cognitive, physiological, and behavioral dimensions of performance anxiety related to musical performance (Kenny, 2009, 2011). An overall MPA score is obtained. The scale shows a reliability scale with Cronbach’s Alpha of 0.91.
- Social Skill Scale, SSS (Gismero, 2010). Two subscales of the SSS have been used: Self-expression in social situations, made up of eight items; and Initiating positive interactions with the opposite sex, made up of five items. In total, 13 items are answered with a Likert scale of 1–4, where 1 is equal to “I do not relate at all; most of the time that does not happen to me or I would not do it” and 4 equals “I strongly agree and I would feel or act like this in most cases.” The subscale Self-expression in social situations reflects the ability to express oneself spontaneously and without anxiety, in different types of social situations. Obtaining a high score indicates ease of interactions, expressing one’s own opinions and feelings, asking questions, etc. The subscale Initiating positive interactions with the opposite sex tries to measure the ability to initiate positive interactions with people of the opposite sex who may be attractive, be it a conversation, asking for a date, spontaneously giving a compliment, etc. A high score indicates ease of such behaviors. Reliability with the global scale Cronbach’s Alpha is 0.88.
- Form for obtaining sociodemographic data. A Google form was used that can be programmed so that none of the questions are left unanswered. As Table 1 shows, the form collected information on gender; current work; musical style; and the musical instrument.

### Procedure

The project was endorsed by the Department of Behavioral Sciences Methodology in the Faculty of Psychology at the National University of Distance Education (UNED in its Spanish acronym). Further, the study was conducted in accordance with the latest declaration of Helsinki (Bošnjak, 2001; Tyebkhan, 2003; World Medical Association [WMA], 2022).

In order to distribute the information among their students and teachers, 1 month before starting the program, they contacted music colleges in different areas of Spain. Details were given of what the program consisted of, how it would be developed and the form to be completed by the participants was attached.

Some personal and sociodemographic data were requested in the form and the three scales were included to evaluate the variables of interest. It was suggested that the completion of the questionnaire evaluating FS should be done after carrying out a public performance (audition or concert) or to respond to this questionnaire considering the last performance or audition performed as a performative situation. In the case of post-test measures, EG responded to the form after carrying out a self-organized public performance (audition or concert) or using a performance planned in their schedule, but under criteria established in the program (see Supplementary Material 2). While CG responded to the post-test form on the same dates as EG after a concert or audition according to the usual course of their schedules.

### Objectives and Contents of the Implemented Program

The Self-Regulation Skills for Performing Musicians (HAMI in its Spanish acronym) program was designed for participants to complete through the online platform Classroom (by Google). A combined approach was used, that is, they carried out the activities independently, but with contact and individualized feedback from the psychologist responsible for the program through the same platform. The program lasted 12 weeks where, on each school day, between 3 and 20 min had to be spent carrying out a task. In absolute terms, there were 60 days in the program.

The program’s objectives were to have a direct impact on each of the components of Flow (see Table 2), and also on related factors (emotional awareness and regulation; interpersonal relationships; values; personal and social well-being; attention; memory; and social support).

**TABLE 2:** Objectives related to components of Flow theory that were intended to be achieved with participation in the implemented program.

Balance	Understand the need for a sufficient level of technical competence in relation to the challenges to be faced.
	Adjust the challenges to personal skills and the situations in which those skills have to be used.
	Transform environments into more challenging ones, deliberately creating an obstacle.
Goals	Set clear goals during study, practice or performance.
	Structure environments to promote different objectives.
	Practice displaying the performance in advance.
	Establish a routine that facilitates reaching the optimal performance experience.
Feedback	Pay attention to your own goals, your own progress, and avoid comparisons.
	Learn to pay attention to the performance.
	Listen to clear feedback to stay tuned to the performance.
	Filter the feedback to keep the valuable information that links to the task.
	Establish a positive and energetic internal dialogue.
Concentration	Organize time to concentrate without disruption to the performance.
	Gradually increase concentration time.
	Learn to listen, observe, evaluate, carefully tune in to the performance.
	Learn to regain focus on the performance.
	Choose and practice the response and reaction that you can have yourself in the face of a distraction, a mistake or any setback.
Merging	Automate skills.
	Learn to pay attention to the body.
	Connect emotion and expression to movement.
Consciousness	Find out what happens to your attention when you become fully immersed in the performance.
	Train your mind in the present moment.
	Pay less attention to your image and the desire to impress.
	Learn to silence everyday issues and concerns.
	Work on and face criticism.
	Foster empathy and positive relationships (to reduce risk).
Control	Learn about the important factors that lead to optimal performance.
	Differentiate between what can and cannot be controlled.
	Create opportunities to display and improve the performance.
	Work on self-confidence.
Autotelic	Recall and reproduce FS experiences.
	Encourage enjoyment as part of the activity.
	Organize practice, study, and commitments so as to avoid burnout.
	Benefit from optimal preparation in different skills, such as technical-performance, mental, psychological, etc.

Different exercises were carried out to develop the self-regulation skills that made up the program’s objective. These exercises were designed from the evidence of different strands of scientific psychology. Specifically, from CBT (Farmer and Chapman, 2016; Gross, 2020), Mindfulness (Shapiro, 2020), Emotion Regulation Therapy (Gross, 2015; Renna et al., 2018), from its own research in Flow Theory (Jackson and Csikszentmihalyi, 1999), States of Optimal Experience (Sinnamon, 2020), and Positive Psychology (Biswas-Diener, 2010; Froh and Parks, 2013; Rashid and Seligman, 2018). The exercises were grouped into four sections (a) emotional and social awareness and regulation, (b) mindfulness exercises, (c) practice and performance preparation exercises, and (d) regulation exercises that, once grasped, are quickly implemented (such as breathing techniques or regulation through the senses) (to see the contents in detail, consult Supplementary Material 2). As mentioned above, each day an exercise was presented, and a total of 60 were performed. They were ordered taking into account the difficulty and were alternated so that exercises from each section appeared regularly.

### Statistical Analysis

To determine the degree of association between the variables, correlational analyses were performed. To test the assumptions of the quasi-experimental design, multi-group analyses were performed with structural equation modeling (SEM) (Holgado-Tello et al., 2016). The models of the FS, MPA, and SS variables were analyzed in order to establish the factorial and measurement invariance between EG and CG in the pre-test measures. The Generalized Least Squares (GLS) estimation method was used. On the other hand, in order to choose the appropriate statistical method to perform the pre-post- and cross-group contrasts, normality tests were performed with significance tests and graphs. Normality assumptions were verified in all the variables. To determine if there were differences between the two groups and between the two temporary measures (pre–post), repeated measures mixed ANOVA and also contrasts for samples related to the Student’s *t*-test were performed. In all cases, the Levene test was performed to verify the assumption of homoscedasticity of the variances of the two groups and was carried out in all the contrasts except those indicated (^a^). Statistical analyses were performed with LISREL 11 (Jöreskog and Sörbom, 2021), PRELIS (Jöreskog and Sörbom, 2021), SPSS for Windows v.25, and G*Power 3.1.9.2 (Erdfelder et al., 1996; Faul et al., 2007).

## Results

### Demographic Analysis of Completer Versus Non-completers

Initially, 91 performing musicians accessed the program. Thirty-one were discarded shortly after starting the program because they did not give any sign of following it, they neither marked the tasks nor established any type of communication with the person in charge of the program. One of the explanations for this behavior would be that they were people interested in seeing the program rather than participating. On the other hand, throughout the program, 32 musicians didn’t follow the pace that had been established to do the tasks and did not finish the program on time. However, it cannot be said that they abandoned it, they remained in the program to do it at their own pace, but they did not enter the investigation. Finally, 28 (EG) participants completed the proposed program and completed the subsequent forms that were considered for data analysis.

A demographic analysis of program completers versus non-completers was conducted to assess their future suitability. Table 3 shows the percentages of participants (completers and non-completers) based on the categorical variables collected. The most relevant data is the highest percentage of completers in the string group (62.5%). In addition, musicians who did not play classical music completed the program in a lower percentage (7.7%).

**TABLE 3:** Percentage of completers (C) versus non-completers (NC) according to the categorial variables considered.

Variable	Categories	% C (*n* = 28)	% NC (*n* = 63)
**Gender**			
	Men (*n* = 32)	28.1%	71.9%
	Women (*n* = 59)	32.2%	67.8%
**Work**			
	Student (*n* = 58)	32.8%	67.2%
	Professor (*n* = 33)	27.3%	72.7%
**Musical style**			
	Classical (*n* = 79)	34.6%	65.4%
	Other (*n* = 12)	7.7%	92.3%
**Musical instrument**			
	Woodwind (*n* = 16)	12.5%	87.5%
	Piano (*n* = 26)	26.9%	73.1%
	Singer (*n* = 18)	22.2%	77.8%
	Strings (*n* = 16)	62.5%	37.5%
	Other (*n* = 15)	33.3%	66.7%

*N = 91.*

### Descriptive Statistics

In general terms, standardized values of skewness and kurtosis out of the range –2 to 2 could be indicating significant deviation from normality (Jöreskog and Sörbom, 1993). The majority of the items presented negative skewness, and all of them were in the range –2 to 2. However, two items (MPA7 and MPA40), presented a high kurtosis.

On the other hand, the Kolmogorov–Smirnov test showed that the EF, AEM, HHSS and the subscales of EF (merging, control, and consciousness), were normally distributed. However, this result was not found in concentration, time and autotelic (see Table 4).

**TABLE 4:** Kolmogorov–Smirnov normality test.

Variables	Mean (*SD*)	*D*	*p*-value
FS	149.79 (39.85)	0.09	0.20
MPA	138.06 (33.92)	0.07	0.20
SS	33.08 (8.18)	0.08	0.20
Merging	24.52 (8.19)	0.10	0.20
Concentration	26.73 (8.27)	0.14	0.00
Control	24.39 (7.77)	0.07	0.20
Consciousness	20.56 (11.00)	0.10	0.20
Time	25.63 (10.61)	0.12	0.04
Autotelic	27.07 (9.58)	0.14	0.00

### Bivariate and Partial Correlations Between Flow State, Musical Performance Anxiety, and Social Skills

Table 5 shows the Pearson correlations between the three dependent variables in the pre-test measures. Correlations between each pair of variables showed statistically significant median associations (*p* < 0.01). The correlation between FS and MPA showed a coefficient of *r* = –0.40; between FS and SS of *r* = 0.45; and MPA and SS of *r* = –0.61. These correlations remained relatively stable with slight variations in the post-test measures (*r* = –0.54, *r* = 0.30, and *r* = –0.55, respectively). Although, if we look at the size of the effect of the correlations (Cohen, 1988), in the pre-test the first two maintained medium effects while the last one had a high effect size. This situation is modified in the post-test, where the correlations between FS and MPA; and between MPA and SS present high effect sizes, while the FS-SS correlation remains at medium levels.

**TABLE 5:** Pearson correlations between FS, MPA, SS (pre-test measures).

	FS	MPA	SS
FS	1		
MPA	–0.395**	1	
SS	0.448**	–0.605**	1

**p < 0.01.

Table 6 shows the pre-and post- partial correlations between each pair of variables, keeping the third controlled. The correlations show that when the impact of SS is controlled, the correlation between FS and MPA decreases with respect to the bivariate correlations (see Table 5) and is no longer statistically significant in the pre-measure (pr = –0.17); however, in the post-measure, the relationship is moderate and statistically significant (pr = –0.47). When the impact of MPA is controlled, the correlation between FS and SS decreases with respect to the bivariate correlations (see Table 5) but remains statistically significant in the pre-measure (pr = 0.29); however, in the post-measure, the relationship between the two variables largely disappears (pr = 0.01). It is possible that practicing exercises aimed at promoting FS and coping with MPA had an effect on EG. Specific components of FS were increased but these effects were independent of SS. Finally, when the FS effect is controlled, the correlations between SS and MPA decrease slightly with respect to the bivariate correlations (see Table 5) and continue to be statistically significant both in the pre- (pr = –0.52) and post- (pr = –0.49).

**TABLE 6:** Partial correlations (pr) in the pre- and post-measures.

Control variable	Primary variables	pr (pre)	pr (post)
FS	SS-MPA	–0.521***	–0.486***
MPA	FS-SS	0.286*	0.005
SS	FS-MPA	–0.174	–0.472***

*p < 0.05; ***p < 0.00.

### Factorial and Measurement Invariance Between Groups

Table 7 shows the overall goodness-of-fit indices of the factor equivalence model and the measurement equivalence between groups in the pre-test measure for the FS, MPA, and SS variables. The values show that the two models, both the equal factor model and the measurement equality model, are invariant between EG and CG in the pre-test measure of FS, MPA, and SS. All overall goodness-of-fit indices show adequate values. That is, we find the same structure in both, and the relationship of each factor with its general factor (FS, MPA, and SS), is equivalent.

**TABLE 7:** Overall goodness-of-fit indices for the multi-group analysis (EG, CG) in the pre-test measure for the Flow, MPA, and SS variables.

Variable	Model	*X* ^2^	df	*p*	ECVI	RMSEA	NNFI	CFI
FS	Invariance factors	16.98	18	0.52	1.10	0	1.11	1
	Measurement invariance	18.08	23	0.75	1.02	0	1.42	1
	*X*^2^ increase	1.1	5	0.95				
MPA	Invariance factors	71.03	70	0.44	2.52	0.02	0.86	0.89
	Measurement invariance	73.45	79	0.66	2.35	0	1.69	1
	X^2^ increase	2.42	9	0.98				
SS	Invariance factors	123.73	130	0.64	3.90	0	1.68	1
	Measurement invariance	141.03	142	0.51	3.70	0	1.10	1
	*X*^2^ increase	17.3	12	0.13				

On the other hand, the values in the chi-square increment were not statistically significant, showing that the saturation matrix of both groups is equivalent in the three variables. Therefore, regarding the equivalence of the control and EGs in the pre-test condition, we have to accept the hypothesis that both groups are invariant.

### Between-Subject Comparisons

The FS, MPA, and SS variables and the six dimensions of FS were analyzed. The ANOVA results in the pairwise comparisons of the between-subject effects tests of the pre-test measures did not show statistically significant differences between EG and CG; except MPA which did show differences [*F*(1,60) = 3.94, *p* = 0.05, η2 = 0.06] (see Table 8).

**TABLE 8:** Descriptive statistics and ANOVA test.

		*M* (*SD*)		*F*	*p*	η^2^	1-β
		EG	CG				
FS	PRE	153.14 (7.57)	147.03 (6.87)	0.36	0.55	0.01	0.09
	POST	171.64 (7.68)	145.32 (6.97)	6.45	0.01	0.10	0.71
MPA	PRE	128.86 (33.00)	145.65 (33.23)	3.94	0.05	0.06	0.50
	POST	117.54 (31.80)	146.38 (34.05)	11.70	0.00	0.16	0.92
SS	PRE	34.14 (7.90)	32.21 (8.41)	0.86	0.36	0.01	0.15
	POST	35.75 (8.04)	32.62 (9.05)	2.03	0.16	0.03	0.29
Merging	PRE	^(a)^25.86 (6.53)	^(a)^23.41 (9.30)	1.38	0.25	0.02	0.21
	POST	28.68 (6.51)	24.53 (8.34)	4.61	0.04	0.07	0.56
Concentration	PRE	27.82 (8.06)	25.82 (8.45)	0.90	0.35	0.02	0.15
	POST	30.21 (7.25)	26.29 (8.22)	3.88	0.05	0.06	0.50
Control	PRE	25.04 (7.86)	23.85 (7.77)	0.35	0.56	0.01	0.09
	POST	28.89 (7.04)	23.38 (9.31)	6.67	0.01	0.10	0.72
Consciousness	PRE	21.14 (11.35)	20.09 (10.85)	0.14	0.71	0.00	0.07
	POST	28.71 (8.28)	18.71 (10.80)	16.18	0.00	0.21	0.98
Time	PRE	24.79 (10.79)	26.32 (10.57)	0.32	0.57	0.01	0.09
	POST	24.36 (11.09)	25.91 (10.86)	0.31	0.58	0.01	0.09
Autotelic	PRE	28.50 (9.00)	27.53 (10.12)	0.16	0.70	0.00	0.07
	POST	30.79 (7.12)	26.50 (10.76)	3.26	0.08	0.05	0.43

*Between-subject factors/pairwise comparison; g.l. = 1.60. Variables: Flow, MPA, SS and the six dimensions of Flow. EG, n = 28; CG, n = 34.*

*M, mean; SD, standard deviation; η^2^, partial eta squared; 1-β, statistical power.*

*^a^Significant Levene’s test.*

In the post-measures, the between-subject effects showed statistically significant differences between CG and EG in the variables FS [*F*(1,60) = 6.45, *p* = 0.01, η2 = 0.10] and MPA [*F*(1,60) = 11.70, *p* = 0.00, η2 = 0.16], but not in SS [*F*(1,60) = 2.03, *p* = 0.16, η2 = 0.03]. In the FS dimensions, there were statistically significant differences between the two groups in the post-merging measures [*F*(1,60) = 4.61, *p* = 0.04, η2 = 0.07], concentration [*F*(1,60) = 3.88, *p* = 0.05, η2 = 0.06] control [*F*(1,60) = 6.67, *p* = 0.01, η2 = 0.10], and consciousness [*F*(1,60) = 16.18, *p* = 0.00, η2 = 0.21]; but not in time [*F*(1,60) = 0.31, *p* = 0.58, η2 = 0.01], nor in autotelic [*F*(1,60) = 3.26, *p* = 0.08, η2 = 0.05] (see Table 8).

### Within-Subject Effects and Comparisons for Related Samples

The ANOVA results in the within-subjects effects tests showed, on the one hand, a statistically significant increase in FS over time (pre-post) [*F*(1,60) = 4.23, *p* = 0.04, η2 = 0.07], and a statistically significant interaction between the group and time [*F*(1,60) = 6,12, *p* = 0.02, η2 = 0.09]. As for MPA, there was a statistically significant decrease over time [*F*(1,60) = 5.56, *p* = 0.02, η2 = 0.09], and a statistically significant interaction between the group and time [*F*(1,60) = 8.71, *p* = 0.01, η2 = 0.13]. Regarding the SS variable, there was no statistically significant difference over time [*F*(1,60) = 3.37, *p* = 0.07, η2 = 0.05], nor was there a statistically significant interaction between the group and time [*F*(1,60) = 1.18, *p* = 0.28, η2 = 0.02] (see Table 9).

**TABLE 9:** Descriptive statistics and ANOVA test.

		M (SD)		*F*	*p*	η^2^	1-β
		Pre	Post				
FS	EG	153.14 (39.23)	171.64 (34.63)	6.12	0.02	0.09	0.68
	CG	147.03 (40.73)	145.32 (44.93)				
MPA	EG	128.86 (33.00)	117.54 (31.80)	5.56	0.02	0.09	0.64
	CG	145.65 (33.23)	146.38 (34.05)				
SS	EG	34.14 (7.90)	35.75 (8.04)	1.18	0.28	0.02	0.19
	CG	32.21 (8.41)	32.62 (9.05)				
merging	EG	^(a)^ 25.86 (6.53)	28.68 (6.51)	0.80	0.37	0.01	0.14
	CG	^(a)^ 23.41 (9.30)	24.53 (7.80)				
concentration	EG	27.82 (8.06)	30.21 (7.25)	1.36	0.25	0.02	0.21
	CG	25.82 (8.45)	26.29 (8.22)				
control	EG	25.04 (7.86)	28.89 (7.04)	6.31	0.02	0.10	0.70
	CG	23.85 (7.77)	23.38 (9.31)				
consciousness	EG	21.14 (11.35)	28.71 (8.28)	12.36	0.00	0.17	0.93
	CG	20.09 (10.85)	18.71 (10.80)				
time	EG	24.79 (10.79)	24.36 (11.09)	0.00	0.99	0.00	0.05
	CG	26.32 (10.57)	25.91 (10.79)				
autotelic	EG	28.50 (9.00)	30.79 (7.12)	2.54	0.12	0.04	0.35
	CG	27.53 (10.14)	26.50 (10.76)				

*Within-subject factors/group-moment interaction; g.l. = 1, 60. Variables: Flow, MPA, SS and the six dimensions of Flow. EG, n = 28; CG, n = 34.*

*M, mean; SD, standard deviation; η^2^, partial eta squared; 1-β, statistical power.*

*^a^Significant Levene’s test.*

Regarding the six dimensions of FS, “merging” showed a statistically significant increase over time [*F*(1,60) = 4.28, *p* = 0.04, η2 = 0.07], however, it did not show a statistically significant interaction between the group and time [*F*(1,60) = 0.80, *p* = 0.37, η2 = 0.01]; in the “concentration” dimension, there was no statistically significant difference over time [*F*(1,60) = 3.02, *p* = 0.09, η2 = 0.05], nor was there a statistically significant interaction between the group and time [*F*(1,60) = 1.36, *p* = 0.25, η2 = 0.02]. Regarding the “control” dimension, it showed a statistically significant increase over time [*F*(1,60) = 3.86, *p* = 0.05, η2 = 0.06], and a statistically significant interaction between the group and time [*F*(1,60) = 6.31, *p* = 0.02, η2 = 0.10]. Regarding the “consciousness” dimension, it showed a statistically significant increase over time [*F*(1,60) = 5.90, *p* = 0.02, η2 = 0.09], and a statistically significant interaction between the group and time [*F*(1,60) = 12.36, *p* = 0.00, η2 = 0.17]. Finally, in the “time” and “autotelic” dimensions, there were no statistically significant differences over time, nor was there a statistically significant interaction between the group and time (see Table 9).

#### Experimental Group

Student’s *t*-tests for related samples showed statistically significant differences before and after the intervention in FS [*t*(27) = –2.41, *p* = 0.02, *d* = 0.36, 1-β = 0.45], MPA [*t*(27) = 2.64, *p* = 0.01, *d* = 0.24, 1-β = 0.24], “control” [*t*(27) = –2.48, *p* = 0.02, *d* = 0.47, 1-β = 0.67], and “consciousness” [*t*(27) = –3.66, *p* = 0.00, *d* = 0.70, 1-β = 0.94]. Therefore, there is sufficient evidence to conclude that the participants in the program showed higher levels of FS and lower levels of MPA after their participation in the program.

#### Control Group

Regarding the CG results, they did not show statistically significant differences in FS [*t*(33) = 0.44, *p* = 0.66, *d* = 0.08, 1-β = 0.07], MPA [*t*(33) = –0.25, *p* = 0.81, *d* = 0.02, 1-β = 0.05], “control” [*t*(33) = –0.52, *p* = 0.61, *d* = 0.09, 1-β = 0.08], nor in “consciousness” [*t*(33) = 0.89, *p* = 0.38, *d* = 0.15, 1-β = 0.14]. Therefore, there is sufficient evidence to accept the fourth hypothesis of the research, which stated that CG will not show differences between the pre-and post-measures of any of the variables considered.

## Discussion

For One of the most common and specific problems for performing musicians is MPA. According to Flow theory, it has been noted that anxiety could have a negative relationship with FS (Csikszentmihalyi, 1975, 1990; Csíkszentmihályi, 1997), which has led to the suggestion that interventions to promote FS could contribute to the reduction of MPA and facilitate musical performance (Lamont, 2012; Wrigley and Emmerson, 2013; Iusca, 2015; Cohen and Bodner, 2019a).

Flow state is a widely researched construct in different fields, especially in the field of Sport Psychology (cf. Jackman et al., 2019). In the field of music, there are more and more studies focused on Flow and related to music education and/or musical performance (Custodero, 2002, 2005; Fritz and Avsec, 2007; Sinnamon et al., 2012; Fullagar et al., 2013; Marin and Bhattacharya, 2013; Wrigley and Emmerson, 2013; Iusca, 2015; Cohen and Bodner, 2019a,b; Moral-Bofill et al., 2020b). In fact, Flow theory provides a framework to promote a more positive and satisfying relationship with performing and preventing MPA.

To that end, the main objective of the present research was to evaluate the effects of an electronically implemented psychological program, aimed at performing musicians, and designed to promote, mainly, the state of Flow, through the development of self-regulation skills and coping with MPA.

To evaluate the results of the intervention program, the factorial and measurement invariance between EG and CG was first analyzed for the three dependent variables in the baseline measurements. The results showed equivalence between the two groups with adequate overall goodness-of-fit indices (see Table 7). This result added greater internal validity to the study since it bases the results on the possible differences between the groups in the baseline measurements of the three dependent variables, and these possible differences can be interpreted without ambiguity (Holgado-Tello et al., 2016).

The results showed that EG and CG were homogeneous in FS, SS, and the six dimensions of FS in baseline measurements, however, the musicians who participated in the intervention had, prior to it, a lower level of MPA than the musicians in CG (see Table 8). This baseline difference in MPA could be related to the fact that the EG musicians were involved in the intervention program for this research because they may have a greater interest in developing self-regulation skills for performance. Therefore, they may have previously been involved in developing these skills independently, or in other programs or processes. It could be that the participants who were involved in the intervention were more interested in doing it because of greater motivation to be performing musicians and this motivation could provoke the need for the musicians to develop psychological skills as well.

Regarding the bivariate correlations between the FS, MPA, and SS variables, the results showed statistically significant median associations between each of them (see Table 5), and they remained stable with slight variations in the post-test measures. As in previous studies (Kirchner et al., 2008; Fullagar et al., 2013; Stocking, 2013; Cohen and Bodner, 2019a; Moral-Bofill et al., 2022), FS and MPA showed a negative correlation (*r* = –0.40) that increased in the post-test measure (*r* = –0.54), strengthening the negative relationship between the two variables. However, in the partial correlations, in the pre-measures, the partial correlation between FS and MPA presented a weak, non-statistically significant association when the effect of SS was eliminated. In addition, the partial correlations showed that each association between each pair of variables was more or less influenced by the effect of a third, depending on the moment in time. In the post-measures varied with respect to the pre-measures, both in FS-MPA and in FS-SS. This result points to the intervention program’s effect on FS-specific and MPA-related components (such as “control” and “consciousness”) but independent of SS, which is reflected in the differences between the partial correlations of the pre-and the post; specifically, in the post-measure’s increase between FS and MPA and, on the other hand, the decrease between FS and SS.

However, the stability of the partial correlation between MPA and SS in the two temporal measures suggests that the development of SS could contribute to reducing general social anxiety and factors of social phobia that, in turn, could be an advantage for coping with MPA, results that, on the other hand, would support studies that highlight the importance of SS in the context of music education and performance (Kemp, 1996; Gaunt and Hallam, 2009; Ascenso et al., 2017; Moral-Bofill et al., 2022).

Regarding the intervention program’s effects on the FS, MPA, and SS levels of the participating musicians, the results showed that the increase that EG had experienced in FS levels was statistically significant, with a small to medium effect size (*d* = 0.36). Similarly, EG experienced a decrease in MPA that was also found to be statistically significant, with a small effect size (*d* = 0.24). It has already been mentioned that EG showed lower MPA levels than CG in the pre-measure. As mentioned above, this result could be related to a greater knowledge of psychological skills that the EG musicians could have developed prior to the intervention. However, although EG had shown lower MPA levels than CG in measures prior to the intervention, those levels decreased even more after the program. On the other hand, CG did not show statistically significant differences between the two temporal measures, neither FS nor MPA. Unlike the study by Cohen and Bodner (2019b), which did not find an improvement in Dispositional Flow measures, the present study did find that activities had an effect on both FS and MPA levels. One of the reasons given by the authors for not finding improvements in Flow was that they used the Flow predisposition scale. In this study, the Spanish validated Flow state scale (Moral-Bofill et al., 2020b) was used. In any case, it is necessary to mention that promoting FS means having an impact on a possible MPA decrease, since some of the components of FS, or components that are conditions for triggering FS, are close to factors related to anxiety, such as consciousness and control, or the need for clear feedback that increases the controllability of a situation. Therefore, MPA is expected to decrease when an intervention is designed to promote FS. In the same way, it is reasonable to think that designing intervention programs to improve MPA should have an impact on FS levels. In fact, the results of this research suggest the same.

Regarding the six components of FS, all pairs of means (pre/post) remained stable in the two temporal measurements in CG (see Table 9). However, the differences between the pre- and post- measures in EG in “control” and “consciousness” were statistically significant and showed a medium effect size for “control” (*d* = 0.47) and large for “consciousness” (*d* = 0.70) (Cohen, 1988). Regarding the rest of the FS components, although the mean values were higher after the intervention in EG in all dimensions (except in “time,” a factor that repeatedly shows weak correlations in the investigations or lack of correlation with the rest of the FS dimensions; Jackson and Eklund, 2002; Fournier et al., 2007; Kawabata et al., 2008; Liu et al., 2012; Wrigley and Emmerson, 2013; Moral-Bofill et al., 2020b), they didn’t show any statistically significant differences. However, the higher score in items of these dimensions as a whole should have contributed to the overall FS scale and the statistically significant increase in FS levels after the intervention. In this sense, intervention programs that affect the emotional, cognitive, and motor aspects (and their interaction) of all dimensions could contribute to increasing the levels of the overall FS scale; for example, experiencing enjoyment inside and outside musical performance activity, improving attention and concentration, automating skills or regulating and expressing emotions in a way that facilitates the performance rather than hindering it, are elements that, in a summative way, could contribute to achieving the Flow experience.

On the other hand, these results could be linked to studies that suggest that FS dimensions could be grouped according to whether they refer to cognitive functions or emotional aspects (Stavrou and Zervas, 2004; Moral-Bofill et al., 2020b). In this regard, a study found that musicians who carried out some type of regular practice to cope with performing (psychological and/or body techniques from different strands of psychology) showed a statistically significant higher level of “concentration,” “control,” and “consciousness” than those who did not carry out any practice for that purpose. In addition, the magnitude of these differences in the measures of “control” and “consciousness” was important, above 0.50 and with good statistical power (>0.80). However, there were no differences in the rest of the dimensions (Moral-Bofill et al., 2022). The “concentration,” “control,” and “consciousness” dimensions would be more related to cognitive aspects, while the “merging,” “time,” and “autotelic” dimensions would reflect the sensations and emotions that arise from the Flow experience. We will return to this point later when the results of the “time” dimension are addressed.

In fact, this research’s intervention program proposed tasks that could directly or indirectly potentially improve the cognitive dimensions; tasks to improve attention and concentration, tasks to regulate thoughts, fears, or self-criticism, tasks to develop self-confidence, emotional regulation exercises, etc. And, transversally, tasks were also proposed that promoted self-care, spending time enjoying various experiences (musical and everyday life), enhancing positive emotions, as well as positive relationships with others. But, as presented in the results, the dimensions that showed statistically significant differences were “control” and “consciousness.” Although it may seem that the tasks intended to increase enjoyment were not effective, in reality, there is a possible explanation. It is difficult to assess whether and in what way these more cross-sectional tasks affected each of the FS dimensions, however, it is now recognized that emotional aspects have an influence on cognitive processes. Cognitive functions are facilitated by positive emotional states (Zull, 2006; Carew and Magsamen, 2010; Dolcos et al., 2011; Jung et al., 2014; Seli et al., 2016; Vogel and Schwabe, 2016; Tyng et al., 2017) (which is why emotional regulation techniques are considered central). Therefore, the benefits of these cross-sectional tasks or the emotional regulation tasks themselves, although not seen directly (for example, in a statistically significant increase in the post-test measure of “autotelic”), could be indirect effects on the cognitive dimensions. In other words, they could have a function of optimizing cognitive functions, in addition to directly influencing a possible increase in the enjoyment of the experience (statistically significant or not).

In any case, the results of this research show that the intervention program had a positive influence on these two FS (and strongly MPA-related) components, that is, on “control” and “consciousness.” It has been suggested that “consciousness” plays an important role in achieving FS. Students who do not self-destructively criticize themselves achieve more Flow than those who have a self-destructive attitude (Kirchner et al., 2008). On the other hand, given the responsibility that performing musicians feel when they perform publicly, the emotional regulation that leads to an optimization of cognitive functions could probably contribute to the perceived control over the activity. And, if they feel in control of the situation and that they are mastering the task, their self-confidence will be boosted. In other words, the confidence one has in one’s own resources to face a situation and achieve a desirable result. Because self-confidence is not a “blind” conviction, such as: “I’m sure it will work out,” “I’m a champion,” etc. but an internal state of psychological strength that implies a real knowledge of the difficulty that you face, one’s own resources that can be used to achieve it, and, based on all that, the realistic possibilities that one has to achieve it (Buceta, 2020). Therefore, self-confidence would be based on the “perceived control” of the situations that are important for the performing musicians; and, in addition, the emotion of enjoyment is more likely to surface. Research on the brain basis of FS in the field of video games showed how activity changes in brain areas that are closely related to emotion and reward processing occurred in response to events characterized by a balance between skill and challenge. These changes in brain activity are a reflection of the rewarding effect of moments when the player masters the challenges of the game (Klasen et al., 2012), that is, when they experience a greater sense of control and mastery of the task.

It is necessary to mention the results of the “time” dimension separately. Of the six dimensions that make up FS, it is the one that showed more stable pre-post-measurements in EG (pre, *m* = 24.79; post, *m* = 24.36). In different research projects, “time” shows weak correlations with the rest of the dimensions (Jackson and Eklund, 2002; Fournier et al., 2007; Kawabata et al., 2008; Liu et al., 2012; Wrigley and Emmerson, 2013; Moral-Bofill et al., 2020b). What had not been found to date is that in an intervention program whose objective was to promote FS, after which the scores increased in five dimensions (statistically significant in two of them), the “time” dimension showed no change and the averages remained the same. It has been suggested that one of the important issues to investigate within Flow theory is whether time transformation is a consistent component of the optimal experience (Abuhamdeh, 2020). Apparently, the results of this research suggest that the “time” dimension may not be part of the Flow experience. However, the fact that it is not a consistent component, that is, it is not affected regularly across different contexts or people while the other dimensions are, or the possible changes in “time” do not occur in association with the rest of the dimensions, may be due to various factors that should be studied (Jackson and Eklund, 2004). In addition, for this research to be fruitful, it would be important to properly operationalize FS (Abuhamdeh, 2020). In the case of performing musicians and regarding the results obtained in this research, a suggestion could be made about the behavior of this variable. On the one hand, performing musicians (such as the participants in this study) who are pursuing higher education or who are active professionals, assume the consequences of their performance with great responsibility and need the optimization of cognitive functions to achieve a good performance level. One of the consequences of measuring FS (as in this study) through a scale with Likert-type responses, prevents detecting the point at which FS would be reached (Abuhamdeh, 2020). What can actually be said is that the particular effects were detected in each FS component. Therefore, it is possible that “time” (as a sensation of the Flow experience) was not affected by the completion of the program because, in reality, that state was not reached, but states close to the Flow experience were. As mentioned above, there is research that suggests that the dimensions of FS can be divided into cognitive (“concentration,” “control,” “consciousness”) and sensory/emotional (“merging,” “time,” “autotelic”). Most likely, sensory/emotional dimensions (such as “time”) may fully or partially emerge as a result of the optimization of cognitive dimensions. When the performing musicians face the performance they try to optimize their concentration, focus on what they are doing, and not worry about any other matter and control the situation. At the same time, they try to feel emotions that facilitate the performance. However, it is likely that, on many occasions, they will be unable to achieve an optimal experience during the activity and fully enjoy it. And yet, their performance is satisfactory and within the standards of a good performance (Sinnamon, 2020). So, in the case of this research, it would have to be said that the most significant effects of the intervention program are that a state of greater sense of control has been achieved, with disregard for what others may think. In addition, there was a more modest increase (statistically insignificant) in the levels of concentration, the sensation of action-awareness fusion, and autotelic experience, but not in the sensation of time transformation. All of this contributed to the fact that there was an increase in the overall FS scale in EG with a statistically significant difference after the intervention. However, it cannot be concluded that the state of Flow was fully achieved.

Regarding the SS variable, the results suggest that the activities that were designed to promote better interpersonal communication, self-confidence in social situations, empathy, and positive feelings toward others, did not cause changes in the SS of the participants (see Table 9). It should be noted that the program was not designed for the development of SS, but some activities that took these skills into account were contemplated. In addition, the implementation of the program coincided with the COVID-19 pandemic situation. Participants may have found fewer opportunities to socialize, interact spontaneously with others, and, ultimately, to best implement tasks aimed at building those skills. A notable fact is that the bivariate correlation between FS and SS (with the total number of participants, *N* = 62) in the post-measure decreased from 0.45 to 0.30; and, in the partial correlation, when the variance that FS and SS shared with MPA was removed, the correlation between the two variables (FS and SS) was basically zero. On one hand, the mean values of these two variables in CG remained the same between the pre- and post-measures (and the differences between them were not statistically significant). Instead, it was EG that showed a statistically significant increase in FS and only a slight non-significant increase in SS.

Therefore, this change in the post-correlation between the two variables (FS and SS) suggests that the FS level increased independently of SS. A future line of study regarding SS would be to study its relationship with FS and MPA through structural equation models that would permit an analysis of the type of effects that occur between these variables.

In summary, results indicated that the intervention significantly improved FS and decreased Music Performance Anxiety of the participants in the EG, but not CG. This suggests that programs whose designs incorporate a combination of all the techniques and methods that were used in the program and that come from scientific psychology could be useful to treat the problem of MPA or to prevent it; and, in addition, they could facilitate FS, greater enjoyment during a performance and potentially better performance quality. The results are consistent with the consideration of integrating various approaches of Psychology for the prevention or treatment of MPA, such as the psychology of emotion and emotional regulation, clinical psychology, performance psychology, and positive psychology (Kaleńska-Rodzaj, 2021).

Furthermore, through the electronic implementation, satisfactory results have been obtained. Although the program, which was carried out with programs whose objective is to treat depression (Andersson and Cuijpers, 2009), anxiety (Penate and Fumero, 2016) or stress management (Frazier et al., 2015; Heber et al., 2016, 2017) is not entirely comparable, the intervention program for the development of self-regulation skills in performing musicians showed efficacy in increasing FS and reducing MPA. One of the features of the programs with computerized treatments for depression and anxiety is that they improved with personalized support (Andersson and Cuijpers, 2009; Penate and Fumero, 2016), and this was a factor that was considered in the design of the program. On the other hand, a significant percentage of the participants in this research were music college students (68%). Although some web-based studies aimed at young people did not find clear evidence of the efficacy of these programs for the treatment of the symptoms of the most common mental disorders such as depression and anxiety (Reyes-Portillo et al., 2014; Taylor et al., 2021), other studies have shown small effect sizes for the treatment of depression, anxiety and stress (Harrer et al., 2019), and medium effects in skills training interventions for mental health prevention (Clarke et al., 2015; Conley et al., 2016). In addition, better results were also obtained when the participants had personalized support (Clarke et al., 2015; Conley et al., 2016).

### Limitations

Limitations and possible future lines of research have already been discussed. But it is necessary to discuss the limitations regarding the generalization of the results because the selected sample is a target population chosen based on some characteristics, not selected randomly. Also, it needs to be emphasized that, the selection procedure was conditioned by the characteristics of working in “life itself” and not working in a research center. In fact, this study, more than usage research, offers the results of the evaluation of the implementation of a self-regulation skills training program. The investigation that was carried out is nothing other than the evaluation of that program. Another limitation is that 63 musicians who entered the program did not participate as planned. As mentioned in the results, about half showed no signs of following it and the other half did so at their own pace. Probably, the first ones were interested in seeing the program, but not doing it. However, in no case did they communicate that they wanted to leave it. In fact, they received the notification to perform the tasks every day until about the middle of the program. On the other hand, 32 musicians freely followed the program and expressed their interest in following it without the pressure of time. Therefore, it is possible that a longer time to complete the program means better results in terms of its completion.

It is also necessary to consider that no performance measures were taken. The pandemic situation complicated communication and collaboration with music institutions in order to take performance measures. But it would be especially interesting to take these measures since another issue that is not yet clear is whether the correlations between performance and FS are due to a good performance generating FS or a FS facilitating performance (Harris et al., 2021). However, this research would have to take on methodological challenges by inevitably posing causality hypotheses from an experimental approach. From the manipulation of factors, clear hypotheses about the processes involved, and direct measurement techniques to assess whether changes in predicted variables mediate the link between FS and performance (Harris et al., 2021).

Regarding the assessment of whether the program’s specific design (including that it was carried out electronically) contributed to the improvements in FS and MPA, it is impossible to say. In order to prove it, it would have been necessary to compare it with other programs (which had been carried out by other groups of participants) and which differed in some element of the design, while controlling the other elements. For example, doing the program by dedicating each school day to carrying out some exercise, compared to doing the program by controlling all other factors, but doing one or two classes a week for the same amount of time. This could be a future line of research that would allow the analysis of whether daily regularity contributes to acquiring self-regulation skills more quickly and efficiently than practice which is more spaced out over time. Lastly, the participants had individualized support and feedback throughout the program (unscheduled). The platform was adjusted so that there was only individual interaction between each participant and the person in charge through private messages. However, the time each participant received from the responsible psychologist was not recorded. Taking this variable into account can improve future research.

### Future Directions

Future research could study whether Internet-based programs with the aim of promoting FS (or in general, providing performing musicians with psychological tools for performance), show similar efficacy compared to face-to-face programs, as well as whether they present a greater (or lower) dropout rate. Or also, to introduce a third possibility in these studies, that is, the design of a program with a combination of the two (electronic and face-to-face). This line of research could be very interesting because elements can be introduced into the design of the electronic intervention program which can be difficult to introduce into group face-to-face student classes (such as more personal and individualized attention or a scheduled daily practice approach). Likewise, the electronic program could also suffer from shortcomings, such as, for example, the hands-on practice of social skills in the classroom with classmates or the close contact (emotional and physical) of the professional in charge of the program. So a combination of the two possibilities (electronic and face-to-face) could be an advantage for the development of these skills. In any case, the presence of these psychological skills development programs in music schools seems necessary for the comprehensive education of performing musicians. Probably one of the most relevant reasons to promote the Flow response in performing musicians would be its relationship with subjective well-being and the quality of the experience during the performance. As long as the institutions and people responsible for educating musicians promote this experience, they will be addressing the need to enjoy the performing activity itself and promoting the mental health of the performing musicians (Moral-Bofill, 2021).

## Data Availability Statement

The original contributions presented in this study are included in the article/Supplementary Material, further inquiries can be directed to the corresponding author.

## Ethics Statement

The studies involving human participants were reviewed and approved by the Department of Behavioral Sciences Methodology in the Faculty of Psychology at the National University of Distance Education (UNED) and were conducted in accordance with the latest declaration of Helsinki (World Medical Association [WMA], 2022). The patients/participants provided their written informed consent to participate in this study.

## Author Contributions

LM-B: conceptualization, methodology, formal analysis, investigation, data curation, and writing – original draft preparation, review and editing. AL and MP-L: methodology, writing-original draft preparation, writing-review and editing, supervision, and project administration. FH-T: methodology, formal analysis, writing – review and editing, and supervision. All authors read and approved the submitted version.

## Conflict of Interest

The authors declare that the research was conducted in the absence of any commercial or financial relationships that could be construed as a potential conflict of interest.

## Publisher’s Note

All claims expressed in this article are solely those of the authors and do not necessarily represent those of their affiliated organizations, or those of the publisher, the editors and the reviewers. Any product that may be evaluated in this article, or claim that may be made by its manufacturer, is not guaranteed or endorsed by the publisher.
