# Supplementary material for: Development of Flow State Self-Regulation Skills and Coping With Musical Performance Anxiety: Design and Evaluation of an Electronically Implemented Psychological Program
Source: Front Psychol. 2022 Jun 17;13:899621. doi: 10.3389/fpsyg.2022.899621 (PMC9248863; doi:10.3389/fpsyg.2022.899621)
Supplement: Supplementary file 2 [file Table_1.DOCX]

Supplementary Material 1

Consent for research and agreement with a declaration of commitment and sincerity to the program.

Please note that by completing and submitting this form you agree to:

1. The use of data for research. You can rest assured that all data will be processed anonymously.

2. Re-complete the form that will come through the classroom platform at the end of the course.

3. The commitment to carrying out the program as planned and being honest when confirming that a task has been done. In the event that you do not have time, or you decide that you do not want to continue, you can always leave the program, without any issue. You only have to notify the person in charge.
