# Supplementary material for: Development of Flow State Self-Regulation Skills and Coping With Musical Performance Anxiety: Design and Evaluation of an Electronically Implemented Psychological Program
Source: Front Psychol. 2022 Jun 17;13:899621. doi: 10.3389/fpsyg.2022.899621 (PMC9248863; doi:10.3389/fpsyg.2022.899621)
Supplement: Supplementary file 3 [file Table_2.DOCX]

Supplementary Material 2

Program development and tasks

## Program development

The program’s contents were organized into four sections that addressed: a) emotional awareness and regulation, b) mindfulness exercises, c) practice and performance preparation, and d) quick self-regulation exercises. The tasks were ordered according to their degree of difficulty and their content. For example, before working with emotional regulation strategies, activities related to emotional awareness were proposed. Or before performing mindfulness techniques that focus on seeing oneself and experiences from a distance, it began with attention to breathing and body exercises.

The program lasted 12 weeks. From Monday to Friday, between 3 and 20 minutes had to be spent on a task each day. Proposals to be carried out during the day were published on the platform at 08:00 (Spanish time). Each task’s explanation stated when to carry it out (throughout the day or at a specific time, for example, at the end of the day). Each task described what to do. Some were accompanied by the necessary material (text, video, or audio) to be able to complete them. Once completed, it had to be marked as done. Thus, a record was kept of who carried it out and at what time.

The platform was programmed to allow interaction between each participant and the person in charge of the program through private messages. Once the participants completed the tasks, they could always re-enter any of them to repeat them. In fact, it was expressly encouraged to perform some tasks on other occasions and contexts (for example, breathing exercises, regulation, emotional awareness, etc.). The last task consisted of carrying out a musical performance under a series of criteria that had been raised during the program in order to control the factors that are considered conditions for achieving FS (balance, goals, and feedback).

## Program tasks organized in the four sections (the order in which they were presented appears in parentheses):

**Emotional awareness and regulation**

Emotional awareness. Paying attention to emotions and identifying them (1)

Awareness of past Flow experiences. Identifying and describing (2)

Awareness of past Flow experiences. Identifying factors that promote and block it (3)

Understanding of emotions and how they progress (10)

Recognition of negative thoughts and personal coping messages (11)

Internal dialogue awareness. Steps to change from negative to positive thinking (12)

Promoting self-esteem through positive self-reflection (13)

Awareness and regulation of the activation level (19)

Increasing experiences of enjoyment in everyday life and in music study and practice environments (22)

Celebrating one’s own achievements (23)

Understanding the action tendencies of emotions (29)

Understanding emotions and their relation to needs (30)

Managing emotions (31)

Awareness of negative bias and investment towards possible positive outcomes (32)

Belief experiments about any concern as musicians (33)

Understanding the comfort zone versus the growth zone (38)

*Empathic and positive relationships with others (39)

Coping with criticism (43)

Making Criticisms (Sandwich Technique) (44)

Distortions of thought. Identification, challenge, evidence to the contrary (50)

Belief Work, ABC Model (51)

Awareness and management of internal criticism (52)

Self-confidence work (55)

**Preparation**

Awareness of one’s ability to maintain concentration (4)

Designing a schedule that plans work/study and leisure (5)

Focusing on feedback that provides valuable information about progress in the activity (14)

Visualization for coping with social situations (15)

Visualization for coping with performing in public (24)

Identifying important feedback to address when studying and acting (26)

Implementing focus (27)

Refocusing on the activity of performing music (35)

Refocusing on the activity of performing music (35)

Planning the public audition according to the established criteria (40):

a) Look for a space that you already know, like, and feel comfortable with (a room, a classroom, a community space, etc.).

b) Possible attendees (acquaintances, colleagues, friends, family, etc.). A minimum of six people. Depending on how the pandemic situation is, it may not even be possible to do it with many more people.

c) Handling the necessary steps to reserve a classroom, a room, a community space, inviting or telling the people who are going to attend, etc.

Planning and practising the repertoire under the following criteria (41):

a) Choosing a repertoire consisting of two to five works that last between 15 and 30 minutes in total.

b) It can be a repertoire scheduled for an audition or performance, a specifically chosen repertoire, or a combination of both possibilities.

c) There should be a balance between the ability that one considers they currently possess and the difficulty of the repertoire. Bearing in mind that the situation of playing in front of an audience is usually a challenge in itself, it can help if the ability outweighs the difficulty a little.

d) Choosing a repertoire that can be mastered by the dates set for the performance. Planning the study, clear objective of how to study in order to resolve technical and performance issues.

e) Focusing on important feedback, which helps to achieve goals.

Gradually practicing performing (recording, informal or family performance, etc.) (45)

Mental practice and memory (46)

Devising and practicing pre-performance routines (47)

Evaluating the performance and preparation plan (48)

Refocusing on the music during the performance and acknowledging any errors in an impartial and non-judgmental way (49)

Practicing focusing on music (53)

Analyzing the factors that may be present in the performance and being aware of which can, and cannot, be controlled (54)

Recognizing and managing intense emotions that accompany performing (56)

Performing and subsequently completing the post-test form (60)

**Mindfulness**

Focusing on breathing (6)

Body scan (16)

Focusing on the walking movement (18)

Focusing on the present moment in daily life (20)

Rest, time to relax, without set goals (25)

Refocus practice (28)

Focusing on movement by executing action patterns by body sections (head, arms, core, legs, etc.) (34)

Mindfulness (42)

Focusing on the present moment in daily life (59)

**Express regulation**

Emotion management guide (7)

Natural breathing (8)

Breathing and emotional intensity (9)

Deep breathing (17)

Fourfold breathing (19)

Concentration breathing (36)

Breathing combined with the inner voice (37)

Regulating activation through the senses (57)

Regulating activation through breathing (58)

*Cross-sectional task, which is a follow-up to other tasks during the completion of the program.
